# Supplementary material for: Inconsistency in the use of the term “validation” in studies reporting the performance of deep learning algorithms in providing diagnosis from medical imaging
Source: PLoS One. 2020 Sep 11;15(9):e0238908. doi: 10.1371/journal.pone.0238908 (PMC7485764; doi:10.1371/journal.pone.0238908)
Supplement: Attachment — Submitted filename: Response to Reviewers.docx [file pone.0238908.s002.docx]

Julian Hong, MD, MS

Academic Editor

*PLOS ONE*

Dear Dr. Hong:

Thank you so much for handling our manuscript. We also would like to thank the reviewers for thoroughly reviewing our manuscript and for providing insightful comments that were helpful in improving the presentation of our study as a whole.

We have revised the manuscript according to the comments as shown below in a point-by-point manner. We have prepared both a clean version and an annotated version of the revised manuscript, the latter of which shows the revisions through the Track Changes function and marginal memos.

We appreciate the opportunity to submit a revision and hope that you and the reviewers will find our revisions to be satisfactory. We look forward to your ultimate decision regarding our work.

Best regards,

Seong Ho Park, MD, PhD

Professor

Department of Radiology, Asan Medical Center,

University of Ulsan College of Medicine, Seoul, South Korea

**Point-by-point responses**

**Reviewer #1:**

The authors are concerned about the varying definitions of the term "validation" in medical imaging AI papers. In the machine learing world, this term usually refers to a dataset used to tune model hyperparameters and decide when to stop training optimization, but in the medical world this term usually refers to the testing of an algorithm on data not used as part of the training process at all. They show that there are many papers that use the term in the first way, and many papers that use it in the second way.

1. The analysis seems to be appropriate and carefully done, and the writing is very clear. In the spirit of the PLOS One data sharing policy, I think the authors need to share their raw dataset now, so the reviewers can better assess the results. There are no anonymity issues that would keep them from sharing this.

**Author response:** Thank you for reviewing our study in detail and providing critical comments. We fully agree with your comment that the raw dataset should be shared. We have included the raw data as supplementary material.

2. It would be helpful to include a table showing a few specific quotes from the 201 papers that illustrate the two uses of the term "validation". For readers not familiar with machine learning, this will help them understand the problem. It would also be interesting to know if all 201 papers included a separate held-out test set, or if some never tested their model on a held-out set (which would be bad).

**Author response:** This is a great suggestion. As per your suggestion, we have added Table 2 to provide example quotes from the papers regarding the terminology usage.

Also, regarding the use of the held-out test set, a small fraction of the 201 papers did not use a held-out dataset for testing and instead evaluated the performance of a complete algorithm using cross-validation methods. We considered the testing step as a stage for assessing the accuracy of a completed algorithm regardless of the nature of the test dataset used. Therefore, we did not limit the testing step to the act of checking the algorithm performance on a held-out dataset, even though the use of a held-out dataset is recommended for testing.

As this new information has been added, we have also revised the statistical analysis section to explore if the use of a held-out test set was associated with the terminology usage. As a result, we found that there was no significant difference between studies that used a held-out test set and those that did not. We added these issues in the revised manuscript, which are indicated by the marginal memos reading “R1-2”.

3. Since it is unlikely the use of the term "validation set" to describe a tuning set will change in the machine learning world, I do not not think medical AI papers will ever have completely standardized terminology. As long as each paper carefully explains its definitions, I do not think it is a major issue. So I am not completely on board with the authors' conclusions about the need for strict guidelines on this topic.

**Author response:** We agree with your comment. We have revised the Discussion section according to the reviewer’s opinion (marginal memos “R1-3”).

Thank you again for reviewing our study in detail and providing critically helpful comments. We hope that our responses and revisions are satisfactory.

**Reviewer #2:**

This paper provides a systematic review and meta-analysis of the term ‘validation’ as used in deep learning studies regarding medical diagnosis. There is a major inconsistency in the medical imaging community regarding the correct use of ‘validation’ in the technical sense. This paper is well written and the topic is important to readers of both PLoS One and the medical imaging community. However, there are several aspects of this paper that I feel need to be addressed prior to publication. My general comments are as follows.

1. First, I don’t believe this is an inconsistency problem (which implies that there is a lack of standard terminology), but rather an inaccuracy problem regarding the correct use of a well-defined, technical term. As the authors correctly point out, the inconsistency of the term ‘validation’ is most likely because the term is used in general communication to refer to testing the accuracy of a completed algorithm. However, the term means something very specific when referring to the science of machine learning, where it is used to define the tuning of hyper-parameters in a model (i.e., validation the training procedure, not the generalization capacity). This definition is canonically accepted, and in general, the term ‘validation’ should not be mis-used in the colloquial sense when referring to technical work. In my opinion, we simply need to educate the medicine community to use the appropriate terminology when applying machine learning approaches, and this confusion won’t be a problem.

**Author response:** Thank you for reviewing our study in detail and providing critical comments. We agree with your comment, and we revised the manuscript accordingly. Also, considering the reviewer’s opinion, we also thought that it would be more reasonable to consider that using the term “validation” for meanings other than tuning, as it is specifically defined in the field of machine learning, is the source of confusion. Therefore, we revised the statistical analysis using the revised binary categorization of tuning alone vs. testing (testing alone or both tuning and testing) instead of the previous categorization of testing alone vs. tuning (tuning alone or both tuning and testing).

The OR was thus re-calculated for using the term to refer to testing (i.e., OR >1 indicating a greater likelihood to use the term to refer to testing in comparison with the reference category), whereas the same was calculated for using the term to refer to tuning in the original version of the manuscript. The updated statistical results showed consistent results with the previous version, although the exact numerical values have been changed. Please refer to the corresponding revised portions of the manuscript, which are indicated by the marginal memos reading “R2-1”.

2. As such, I do not agree with lines 204-206, where the authors argue, “It would be ideal if medical journals would unify the use of the term validation to refer to to the testing step instead of the tuning step”. In my opinion, there is nothing to ‘unify’ here, and instead the medical journals need to enforce the correct use of terminology. If research related to machine learning (including deep learning) is to be published in scientific journals (including imaging journals), it should use canonical terminology. Superficially changing these technical terms to colloquial terms would only cause more confusion, and question sound scientific reasoning published in the journals.

**Author response:** We agree with your comment on how the medical community should be more aware of the correct use of said terminology. We have revised a paragraph in the Discussion section accordingly (marginal memo “R2-2”).

3. Next, while I find it interesting that papers published in high impact factor journals tend to use ‘validation’ to refer to model testing (instead of the more appropriate and accurate model tuning), I don’t think this statistic should be used as a rationale to justify the proposal in Lines 206-207 as stated above. If anything, it makes more sense to test if there was a statistically significant difference in use of ‘validation’ between technical machine learning journals and imaging journals, regardless of impact factor.
**Author response:** We agree that the statistical results should not be used as a rationale to justify the proposal as stated above. We have revised the Discussion accordingly (marginal memos “R2-3”).

4. The authors argue at Lines 206-207, “Such a unification in terminology use may be difficult in disciplines such as machine learning, where the term is relatively widely used to refer to the tuning step”. This line similarly does not make much sense to me; machine learning is the discipline being used, regardless of the application (i.e., medical imaging or otherwise). In the context of this paper, it is all machine learning, and imaging is simply the application. It is not a one-to-one fair comparison.

**Author response:** We agree with your comment. We have revised the Discussion section accordingly (marginal memo “R2-4”).

5. On Lines 219-236, the authors discuss “internal validation vs. external validation”. Here, they are using the term in the colloquial sense, but in the paragraphs that follow, they actually explain a process that is technically already defined as “internal testing vs. external testing”. By definition, a model that has been validated on an internal dataset (i.e., the learning procedure has been (cross)-validated to identify optimal hyper-parameters) has to be tested on a separate dataset not included in the original training and validation procedure, either internally (on a subset of the dataset held out) or externally (on a completely independent dataset). The notion of “internal validation vs. external validation” is ill-defined and goes against the entire purpose of this paper.

**Author response:** As per your comment, we have revised the terms to “internal testing” and “external testing”. Thank you for the keen comment.

6. In technical machine learning workflows, it is often implied that model validation is based on some form of a cross-validation procedure, where the the training of a machine learning algorithm is applied to sub-sets (e.g., folds) of the data set to best learn the optimal hyper parameters. The authors to not mention this at all, and it is important as it may help to better define and impairment proper terminology.

**Author response:** We have revised the corresponding portions as suggested.

7. Finally, I think that the Discussion section needs to be substantially expanded on, to include specific examples that are related to imaging. Further, I would like to see the authors also take more of a stance on promoting the appropriate use of the term ‘validation’, as stated in the above points. While the Discussion section does a nice job at documenting the different uses of the term ‘validation’, in my opinion it falls short in promoting a good solution, and should focus primarily on well-defined definitions as a reference point.

**Author response:** We have newly added Table 2 to show the specific examples from the reference papers, and revised the Discussion in multiple locations to incorporate the points raised by the reviewer (marginal memo “R2-7”).

Again, we very much appreciate your insightful inputs. We hope our revisions are satisfactory.
